# Voices of Musicians: Virtual Live Bedside Music Concerts in Inpatient Care

**DOI:** 10.3390/healthcare11222929

**Published:** 2023-11-09

**Authors:** Melanie Ambler, Andrew Janss, Randall S. Stafford, Bryant Lin, Aubrey Florom-Smith, Augustine W. Kang

**Affiliations:** 1 Stanford University School of Medicine, Stanford, CA 94305, USA; 2 Project: Music Heals Us, Guilford, CT 06437, USA; 3 Brown University School of Public Health, Providence, RI 02912, USA

**Keywords:** music, music-based interventions, complementary therapies

## Abstract

The COVID-19 pandemic presented unprecedented challenges to patients, family members, and healthcare staff that resulted in increased stress and isolation and decreased quality of life. We evaluate the impact of a novel virtual concert program, the Vital Sounds Initiative (VSI) of Project: Music Heals Us (PMHU), which began at the beginning of the pandemic to combat patient isolation and provide employment to professional musicians. Using a qualitative analysis of VSI data, we examined post-concert written responses by musicians. These responses were coded by independent coders via inductive coding and thematic analysis. Between 7 April 2020 and 20 July 2022, 192 musicians played 2203 h of music for 11,222 audience members in 39 care facilities nationwide. A total of 114 musicians submitted a total of 658 responses. Three main themes (with corresponding subthemes) arose: (1) Patient Experience; (2) Musician Experience; (3) Caregiver (family or staff) Experience. The responses offered valuable insight into the overwhelmingly positive aspects of the virtual concerts. Overall, we found that VSI favorably impacts individuals at every level, including the patients, musician, and caregivers. These findings provide preliminary evidence for the benefits of virtual music concerts. Upscaling similar virtual music interventions/programs should be considered.

## 1. Introduction

At the height of the COVID-19 pandemic, hospital visitation was strictly limited to curtail the spread of infection. As a result, there were increased reports of distress, grief, and a reduced quality of life in both patients and family members [1]. The restrictions and changes in work routines also negatively impacted healthcare providers, resulting in decreased well-being as well as symptoms of compassion fatigue and burnout [2,3,4].

Not only were individuals within the medical field affected by the pandemic, but so were professional musicians. In a study looking at musician emotional well-being during May–June 2020 of the COVID-19 pandemic, Whitley et al. found that musicians experienced a lack of performance opportunities, cancellations, a lack of access to teachers and students, and reduced income [5]. Virtual performance became one strategy for musicians to mitigate these adverse psychological and material impacts of the pandemic.

The Vital Sounds Initiative was founded by the non-profit organization Project: Music Heals Us (PMHU) at the start of the COVID-19 pandemic to provide live virtual concerts by professionally trained musicians for critically ill patients (in the intensive care unit), family members, and hospital staff. It has since expanded to reach other patient populations within rehabilitation centers, transfusion centers, and memory care facilities. It is the first of its kind in its approach and provides a virtual, personalized musical performance by a professional musician with a special focus on reaching isolated patients who do not have access to traditional in-person art and music performances. To document the unique impact of the VSI of PMHU, we asked musicians to reflect on their experiences, which form the focus of this report. 

The VSI of PMHU aimed to solve two consequences of the pandemic: the increased isolation and reduced quality of life experienced by hospitalized patients and the hampered opportunities faced by musicians. By linking hospitals and their patients with musicians able to provide virtual performances, the initiative sought to improve patient quality of life through a new mode of delivering income-generating musical performances. 

Music listening as it applies to the biopsychosocial model of health has been shown to impact the psychobiological stress system by decreasing cortisol levels in those listening to music that experience a stressor [6]. Additional studies have shown music to have calming effects, reducing stress and anxiety through activation of the parasympathetic nervous system and decreased activity of the sympathetic nervous system [7,8]. Exposure to patient-preferred music in the perioperative setting, for example, has been found to reduce anxiety, relieve post-operative pain, improve patient satisfaction, and regulate hemodynamic parameters [9,10]. With significantly heightened levels of stress, anxiety, and depression reported in patients hospitalized with COVID-19 [11,12], music listening interventions became of interest in reducing these symptoms.

Recent studies have indicated the increased prevalence and role of virtual interventions due to the COVID-19 pandemic on establishing agency and community [13]. To combat isolation, stress, grief, and other emotional consequences of the COVID-19 pandemic, novel virtual music programs such as the VSI of PMHU were offered. Initial case study findings suggested that individualized virtual concerts maximized participant and musician engagement and improved feelings of connectedness. The benefits extended beyond the patient to the staff and musicians themselves [14]. One study pointed to the positive impacts of a single, live music performance session for patients with COVID-19 in significantly reducing anxiety and improving oxygen saturation [15]. We sought to examine the benefits that virtual interventions such as the VSI of PMHU may provide, given their ability to be implemented on a large, accessible scale.

This study aims to elucidate the impact of virtual concerts on patients, musicians, staff members, and loved ones through a qualitative analysis of musician responses collected after each concert. The use of virtual concerts is a novel approach to providing accessible music services to patients. Identification of the most important themes served as a method of improving the structure of the concerts and identifying potential outcomes of interest for a quantitative pilot study to further investigate the effects of these concerts.

## 2. Materials and Methods

We examined the survey responses of musicians participating in the VSI as part of a quality-improvement project by its founding organization, Project: Music Heals Us, a United States-based nonprofit organization. The basic structure of the virtual concert is as follows: a professional musician is assigned to a two-hour block of time with a specific department in a partner healthcare facility. During this time, the staff identifies patients that would like music, and the musician performs a personalized concert for the patient. While there are no hard inclusion or exclusion criteria for patients to qualify to receive a virtual concert, facilitators are encouraged to direct these interventions towards patients experiencing psychophysiological issues related to feelings of isolation. Patients consent verbally, except for intubated COVID-19 patients, whose consent is obtained from their next of kin. Recruitment processes differ at each institution based on the facilitator’s position and bandwidth, and the best practices for patient identification and recruitment need further study and refinement. Concerts vary in duration and style (it is dependent on the musician/the flow of the healthcare facility). 

In addition, concerts are performed via two-way video (camera and audio are on for both the patient and the musician), one-way video (the patient can see and hear the musician, but the musician cannot hear or see the patient), or no video (live audio only), with the vast majority being performed via two-way video. Music therapists were not involved in the study design. Musicians were encouraged to use external microphones for optimized sound quality. In the case that musicians did not have a dedicated external microphone, they were provided a “best practices” information sheet for optimizing the audio settings to provide the best quality sound. Each site had a different mode of connecting to the concerts and was encouraged to use an external speaker with their concert tablets to enhance sound fidelity, though lack of availability or funding for such equipment was not a disqualifying factor.

**Figure 1:**
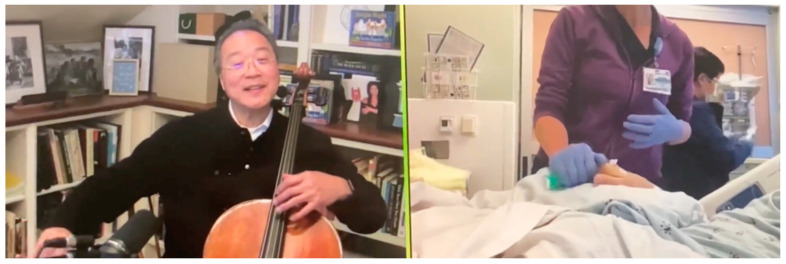
Renowned cellist Yo-Yo Ma performs for a patient and staff member during a VSI concert.

To track the concerts, musicians filled out an online form after each concert session. On this form, there is an optional space to write any “notable or positive reactions by patients or staff”. Patients ranged from being in the COVID unit to the intensive care unit (ICU), general floor, memory care, palliative care, labor and delivery, cancer center, transplant service, and neurological rehabilitation (Figure 1). Musicians were given the freedom to play songs from their own repertoire, or as requested by patients. As such, a range of music genres was offered to patients depending on the musician assigned to them, from classical to jazz to pop. 

Principles of thematic analysis were used to identify codes in the responses. Two coders independently coded the responses and subsequently discussed discrepancies in their coding. The coders adopted an iterative approach to apply code names to quotes, and conflicts and/or discrepancies were arbitrated by a third coder to ensure the validity of the analysis. A code was finalized for inclusion if it had at least three example quotes from three separate participants. A codebook was subsequently generated. Once each individual code was identified, a sub-theme was created to group similar codes (in terms of semantics and topic/subject matter) together, and then subsequent overarching themes were named. To aid in data organization and coding, the qualitative analysis software NVivo 14 was used. No patient data or information were collected. Other collected data included minutes played, hospital locations, and type of musical instrument. Frequencies and descriptive statistics were examined and discussed. 

## 3. Results

### 3.1. Study Population

Between 7 April 2020 and 20 July 2022, 192 unique musicians played 2203 h of concerts to 11,222 audience members (patients, staff, and family members) in 31 hospitals and eight other healthcare facilities (such as infusion centers or nursing homes) nationwide. The median number of minutes played per 2 h “on call session” was 60 min (IQR 42–120 min), with a median of five audience members (IQR 3–9 people) per session. A total of 114 musicians wrote 658 open-ended responses regarding any notable reactions they received from patients or staff. Of these 11,222 musical interactions, 52.8% were given by males, 45.9% by females, 2.0% were non-identified, and 0.3% were given by a mixed-gender group. The most common instrumentation for the concerts included piano (24.8%), violin (19.1%), cello (12.7%), viola (8.7%), guitar (8.5%), and harp (7.5%), with other instruments or groups accounting for the remaining 18.7% of performances. The top five geographic locations were Los Angeles, California; San Diego, California; Ann Arbor, Michigan; Rochester, New York; and New Orleans, Louisiana.

### 3.2. Overview of Thematic Findings

**Figure 2:**
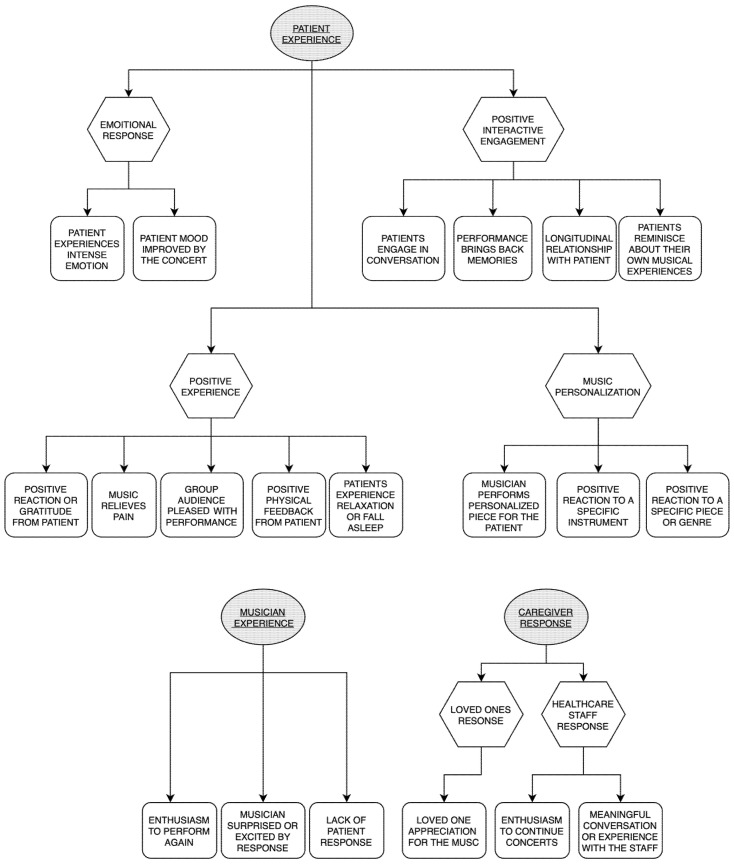
Summary of themes, sub-themes, and codes.

Figure 2 summarizes the results of the study by themes (i.e., a broad-level categorization of codes), sub-themes (i.e., a secondary organization of themes, intermediary between themes and codes), and codes (i.e., the label of a collection of quotes with a unifying semantic). The following themes are described: (1) Patient Experience, (2) Caregiver Experience, and (3) Musician Experience. Each theme is further subdivided into sub-themes and codes with a representative quote from each code. There were 842 individual phrases from the 658 open-ended responses that were coded into themes. The percentage of total responses for each code is included with each code title below.

#### 3.2.1. Theme 1: Patient Experience

The theme of Patient Experience corresponded to any reaction the patient specifically demonstrated to the musician either verbally or non-verbally.

i. Emotional Response

Patients often expressed an emotional response to the music through comments or facial/body language.

Patient experiences intense emotion such as crying or verbal expression (22/842, 2.6%): “*A patient was in tears and thanking me profusely for my playing and for visiting. It was quite humbling*”.

Patient mood improved by the concert (14/842, 1.7%): “*Music gives you a good life, makes you think about a lot of things … this is very nice and gives me good spirits, you have made me happy*”.

ii. Positive Interactive Engagement

Musicians reported conversations and engagements with patients that left a lasting impact on them, suggesting an increased feeling of belonging and decreased isolation. Patients often shared stories from their lives or their own experiences with music.

Patients engage in conversation (60/842, 7.1%): “*Another patient requested to see me today just to talk with me. I’ve played for him many times in the past, but today he was not feeling up to it. But he’s being moved to a new floor soon, so he wanted to say goodbye and that he really enjoyed the music*”.

Performance brings back memories (17/842, 2.0%): “*After I played a cover of, ‘Hey Jude’, by the Beatles, a patient in neuro rehab recalled to me having heard that song as the first song on the radio after they gave birth to their second daughter. They said the music brought them back to the first time they held their child*”.

Longitudinal relationship with patient (13/842, 1.5%): “*I played for a patient for the second time during this visit. She remembered me from last week and was very happy to share the news that she is leaving the hospital soon. She enjoyed talking about music, my instrument, and my future*”.

Patients reminisce about their own musical experiences (24/842, 2.9%): “*One patient cried softly throughout my visit with him because the pieces I played were ones that he used to play on the piano 70 years ago*”.

iii. Positive Experience (according to musicians)

According to the musicians, patients overwhelmingly responded positively to the virtual concerts often via expressions of gratitude, physical feedback, or signs of relaxation.

Positive reaction or gratitude from the patient (292/842, 34.7%): “*One patient commented that she could listen to the music all day and that she didn’t want to share the iPad with others because she was enjoying herself so much*”.

Music relieves pain (5/842, 0.6%): “*I was in considerable pain for a few hours, and this has helped quite a bit!*”

Group audience pleased with performance (32/842, 3.8%): “*I felt great attention from the audience during and after my performance and enjoyed answering their questions and very kind comments!*”

Positive physical feedback (clapping, smiling, singing along, dancing, tapping fingers) (77/842, 9.9%): “*Residents with a significant degree of dementia who are alert but generally non-verbal and relatively inactive were moving/tapping their fingers”*.

Patients experience relaxation or fall asleep (54/842, 6.4%): “*Today I had a nurse tell me (after a specific room as she was wheeling me on the iPad to another room) that the patient I worked with had dementia, and he had been restless, agitated, upset, and combative that day. She said that when he heard me singing and heard the ‘pretty music’ that that was the calmest and most at peace he had been*”.

iv. Music Personalization

In a majority of the performances, musicians performed a piece of music that was requested by the patient. Musicians also reported meaningful responses to their specific instrument or a genre of music that they chose to play.

Musician performs personalized piece for the patient (54/842, 6.4%): “*A patient and his wife cried after I played the song that summed up their life when they were dating*”.

Positive reaction to a specific instrument (14/842, 1.7%): “*The second patient really enjoyed hearing the harp and kept encouraging me to play with their words and clapping and smiling*”.

Positive reaction to a specific piece or genre (52/842, 6.2%): “*One patient was entirely motionless and quiet during the first few songs I played, even after trying to engage with him. But then one of the OTs said, ‘you like Amazing Grace, right?’ and affirmed that. After I played that, I asked him if he had any other hymns he liked. This led him to tell me about the first time he crossed the Atlantic on a ship: there was a pianist on the ship that could play anything you sang. This patient started to sing a hymn to see if I could recreate that same phenomenon. The whole time after this, we got the patient to sing and enjoy all his favorite hymns. He was a completely transformed person, and it was all because we found a genre of music that deeply resonated with him*”.

#### 3.2.2. Theme 2: Caregiver Experience

In their responses, musicians often discussed the impact that the virtual concerts had both on the staff members and the loved ones of the patients.

i. Loved One Response

Loved ones of the patient (family or friends) were present for the concert session and relayed to the musician their appreciation for the concert, and/or their opinion of the effect it had on the patient.

Loved one’s appreciation for the music (21/842, 2.5%): “The husband of another patient relayed a story about his wife, that she took many years of piano lessons as a child. At her high school senior recital, she played a piece whose name the husband couldn’t remember (the wife couldn’t speak because of her stroke). But he gave the impression of the piece with a scatted version. I made an educated guess which piece he could mean, so I said, here’s the first movement of Beethoven’s Pathétique Sonata. And when I started to play the first line, the husband exclaimed, ‘Son of a b***h, that’s it!’ After I finished, he said his wife was playing in the air”.

“*I also played for a couple who was having a blast with me today. I happened to play Sweet Caroline and the wife came onto the screen and said that’s her favorite song because it’s after her namesake*”.

ii. Staff Response

Staff members were often present for the concerts, facilitating group or individual concerts for patients, floating in and out to assure that the technology was functioning properly.

Enthusiasm to continue the concerts (5/842, 0.6%): “*I look forward to this every week—this will lift my spirits for the whole day. I haven’t seen my patients smile like that in such a while, I feel like I have the best job in the hospital today*”.

Meaningful conversation or experience with the staff (55/842, 6.5%): “*One really cool moment was playing for one of the social workers on the floor. I had been playing for her for a few minutes and just decided to play Somewhere Over the Rainbow next. After I finished, she showed me the piece of paper with that same song written on it because she was going to request right before I started playing it. It was a very serendipitous moment! She teared up twice because the music reminded her of her grandma*”.

#### 3.2.3. Theme 3: Musician Experience

Certain themes specifically demonstrated reflection on the part of the musician regarding the impact performing the concerts had on them.

Enthusiasm to perform again (3/842, 0.4%): “*This is definitely the best part of my day. I look forward to this every week*”.

Musician surprised or excited by response (23/842, 2.7%): “*It’s been an incredibly moving, fulfilling, and freeing experience to be able to use my voice in this way. Singing for a growing number and wide variety of patients, I’ve appreciated the opportunity to see the breadth of people, identities, and personalities, and refine how I can learn and respond to what they each want. It’s also been an enjoyable challenge of changing musical styles and repertoire on a dime*”.

Lack of patient response (5/842, 0.6%): “Some patients are “*unresponsive” or “non-verbal”, and one can only hope they are getting some enjoyment out of the music*”.

## 4. Discussion

Our findings indicate that virtual, live bedside music concert programs for patients result in positive experiences that extend beyond the patient to the musician, family members, and hospital staff. Our study also contributes significantly to the literature by documenting the unique perspective of the musicians, who are key change agents in the VSI program. Given the breadth of hospital services, locations, and instrumentation, we show that virtual concerts may feasibly be implemented in a positive manner. 

Past studies have focused on examining the impact of music on its recipients (patients). Music has a positive impact on patients’ well-being and recovery in a variety of clinical settings. A systematic review and meta-analysis by Bradt et al. found that music interventions, including music listening, were associated with significant reductions in anxiety and pain in hospital settings [16]. Another systematic review by Koelsch (2014) found that music can also have positive effects on cognitive and motor function in patients with neurological conditions such as stroke and Parkinson’s disease [17]. Our study, on the other hand, contributes uniquely to the literature by examining the VSI through the musicians’ lenses. Reassuringly, our results are consistent with studies indicating that music can be an effective intervention for improving patient well-being in clinical settings. In particular, the ability to personalize improvisational music may increase the effectiveness of music-based interventions by increasing patient engagement [18,19].

Our findings document the potential positive impact of the VSI on caregivers, which offers an interesting perspective for research examining caregiver support, as most interventions/programs targeting caregivers involve the provision of education and/or training to caregivers (so that they may better provide care and support to the patient), rather than a passive “diffuse” benefit such as via listening to music being played to patients [19]. Caregiver involvement can have a positive impact on patient outcomes, as they provide emotional/instrumental support, help with communication, and assist with decision-making [20]. However, caregivers may also experience stress and burden because of their involvement, so it may be important to provide some form of support and resources to them as well.

Our findings document several important process evaluation findings. Firstly, we report enthusiastic support for the VSI by hospital staff, (i.e., physicians, nurses, and administrators) who are key enablers of the program. A novel program such as the VSI requires programmatic administrative support to be effective, and the assuring findings of staff support may bode well for the upscaling of the VSI as a larger-scale program. In addition, we also document positive and enthusiastic experiences from musicians, who relate that performing for patients can be an engaging and meaningful undertaking. Our results, however, also uncovered potential areas of improvement for music-based interventions. From the musicians’ perspective, playing to patients who may not be able to communicate (e.g., non-verbal patients) or who prefer to have their cameras turned off may be an unsatisfying experience. One limitation of the current study is that most of the concerts given were two-way video calls with minimal one-way or audio-only calls; hence, we are unable to ascertain the importance of video modality for all involved in the concerts.

Our study also raises important unanswered questions warranting further study. For example, some musicians reported the use of a chat function to communicate with patients unwilling to use their camera or microphone. Hence, further research is necessary to examine how best to maintain/improve musicians’ engagement levels for varying patient acuities. We also recommend that future research considers the potential differences in impact via modality of musical performance delivery (e.g., live vs. recorded playlists). Furthermore, future research must rigorously quantify the benefits of programs like the VSI to patients, staff, and musicians via robust scientific methodology, such as clinical trials. Doing so will also identify key mechanisms by which music impacts outcomes, as is recommended by previous research [21].

While virtual musical performance on the scale implemented by the VSI is new, this technological adaptation is not. The rapid adoption of this musical delivery model is one of several examples where the COVID-19 pandemic has greatly accelerated the adoption of previously known but underused processes. 

## 5. Conclusions

In summary, our results document overwhelmingly positive comments from musicians participating in the VSI. Limitations of our study design (cross-sectional, survey-style open-ended responses) need to be addressed with future studies that conduct in-depth qualitative interviews with musicians to better understand the nuances of their experiences playing for the VSI. Regardless, to the authors’ knowledge, this study is the first to present musicians’ perspectives of their experiences with participating in a music-based program for patients and provides vital preliminary evidence supporting the upscaling and widespread adoption of virtual concerts in the hospital setting.

## Data Availability

Data available on request due to ethical restrictions. The data presented in this study are available on request from the corresponding author.
